# Reliability and Validity of the Japanese Version of the eHealth Literacy Scale in Community-Dwelling Older Adults: A Cross-Sectional Study

**DOI:** 10.3390/ejihpe16010001

**Published:** 2025-12-19

**Authors:** Takehiko Tsujimoto, Takafumi Abe, Yoko Kuroda, Masayuki Yamasaki, Minoru Isomura

**Affiliations:** 1 Faculty of Human Sciences, Shimane University, 1060 Nishikawatsu-cho, Matsue City 690-8504, Shimane, Japan; myamasak@hmn.shimane-u.ac.jp (M.Y.); isomura@hmn.shimane-u.ac.jp (M.I.); 2 Center for Community-Based Healthcare Research and Education (CoHRE), Head Office for Research and Academic Information, Shimane University, 223-8 Enya-cho, Izumo City 693-8501, Shimane, Japan; t-abe@med.shimane-u.ac.jp; 3 Department of Neurology, Faculty of Medicine, Shimane University, 89-1 Enya-cho, Izumo City 693-8501, Shimane, Japan; kurodayo@med.shimane-u.ac.jp

**Keywords:** digital health literacy, eHealth Literacy Scale, older adults, validity, reliability

## Abstract

The Japanese version of the eHealth Literacy Scale (J-eHEALS) measure has primarily been applied to younger populations; however, the psychometric properties of the J-eHEALS in older adults have not been investigated. Therefore, in this cross-sectional study, we aimed to evaluate the psychometric properties of the J-eHEALS in community-dwelling older adults. A total of 553 adults aged ≥ 65 years (mean age, 73.5 years) attending routine health checkups in a single Japanese municipality completed the J-eHEALS and the Japanese version of the 12-item Health Literacy Scale (J-HLS-Q12). We examined internal consistency, item characteristics, factorial validity using exploratory and confirmatory factor analyses, measurement invariance by sex, and convergent and criterion-related validity with general health literacy. The J-eHEALS scores indicated moderate to slightly low perceived eHealth literacy in this population. The scale demonstrated excellent internal consistency (Cronbach’s α = 0.94), a stable unidimensional factor structure with acceptable model fit across sexes, and moderate positive associations with general health literacy. Overall, these findings support the J-eHEALS as a reliable and valid instrument for assessing perceived eHealth literacy in older Japanese adults and its suitability for use in research and practice.

## 1. Introduction

The development of Internet infrastructure and rapid dissemination of digital devices (e.g., personal computers, smartphones) over the past three decades have enabled people to access a vast amount of information. Currently, the Internet is considered one of the most important sources of health-related information. In Japan, national surveys revealed that Internet usage reached approximately 90% among individuals in their 60s and approximately 70% among those in their 70s, with Internet usage rates continuing to increase annually (Ministry of Internal Affairs and Communications, 2025). Another survey reported that approximately 60% of Japanese adults aged ≥65 years accessed medical and health information through their personal computers or smartphones (Cabinet Office, 2023). These trends suggest that older adults have greater access to digital resources and are increasingly likely to rely on online platforms when making health-related decisions.

People sometimes obtain medical and health information online before visiting healthcare institutions or as an alternative to professional consultation (Popovac & Roomaney, 2022). Nevertheless, the quality of such information varies considerably, with some content being commercially motivated or unreliable. Online medical and health information comprises a mixture of accurate resources and poor-quality or misleading content (Zhang & Kim, 2022). Thus, the ability to search for, collect, evaluate, and apply online health information to one’s own health problems has become essential; this competency is referred to as “eHealth literacy” (eHL) (Norman & Skinner, 2006a, 2006b). eHL has been demonstrated by empirical studies to be associated with physical activity and dietary behaviors (K. Kim et al., 2023; Mitsutake et al., 2016), self-care practices (Bin Naeem et al., 2024), and health information utilization (Liu et al., 2024). Because older adults are often late adopters of Internet technologies, their eHL scores tend to be lower than those of younger populations (Cao et al., 2023), raising concerns about disparities in digital information.

Norman and Skinner (2006a) developed the eHealth Literacy Scale (eHEALS) to measure eHL, and Mitsutake et al. (2011) created a Japanese version of this scale. The Japanese version (J-eHEALS) had been validated by an Internet-based survey targeting individuals with a mean age of 39.6 years (standard deviation [SD]: 10.9). This relatively young sample likely overrepresented adults who are already comfortable with digital technologies. The J-eHEALS measures have primarily been applied to younger populations, such as female high school students (Takeda et al., 2025) and adults aged 20–59 years in Internet-based surveys examining eHL and cancer-related knowledge (Mitsutake et al., 2012). These studies have advanced understanding of eHL levels and their correlates in non-older populations; however, they did not investigate the psychometric properties of the J-eHEALS in older adults. Older adults differ from younger groups with respect to not only their familiarity with online platforms but also their cognitive and behavioral approaches to health information. Whether the J-eHEALS accurately reflects eHL in older populations remains unclear; therefore, empirical investigations with an older sample are necessary.

Establishing a reliable and valid measurement tool for older adults is crucial for informing public health strategies in Japan, where population aging is rapid. Accordingly, the present study aimed to evaluate the reliability (internal consistency) and factorial validity of the J-eHEALS in community-dwelling older adults.

## 2. Materials and Methods

### 2.1. Study Design and Sampling

In this cross-sectional study, data obtained from participants who underwent routine health checkups (including specific health checkups for individuals aged < 75 years and health checkups for older adults aged ≥ 75 years) in Unnan City, Shimane Prefecture, Japan, were analyzed. This municipality was selected because the local government has an established collaboration with our research team for data collection in community health checkups, which enabled feasible recruitment of community-dwelling older adults in a defined area. Data were collected from August to September in both 2023 (n = 893) and 2024 (n = 823) to maximize the number of participants, as attendance at routine health checkups varies from year to year. Some individuals attended the health checkups in both years; to avoid duplicate observations, we followed our pre-specified analysis plan and retained only the data from each participant’s first health checkup during the study period. Consequently, the analytic dataset comprised 1168 unique participants across the 2 survey years. Among these, the data from 553 participants aged ≥65 years who provided complete responses to the J-eHEALS and Japanese version of the 12-item Health Literacy Scale (J-HLS-Q12) were analyzed.

The study protocol was reviewed and approved by the Medical Research Ethics Committee of the Shimane University Faculty of Medicine (approval number: 20051214-3; approval date: 30 March 2022). Prior to participation, written informed consent was obtained from all participants after receiving explanations about the study.

### 2.2. Measurements

#### 2.2.1. J-eHEALS

eHL was measured using the J-eHEALS, which demonstrated good internal consistency (Cronbach’s α = 0.84) in a previous validation study (Mitsutake et al., 2011). The J-eHEALS comprises eight items evaluating an individual’s perceived skills in locating, evaluating, and applying health information obtained from the Internet. Each item is rated on a 5-point Likert scale ranging from 1 (strongly disagree) to 5 (strongly agree). The total score is calculated by summing the scores of all 8 items, resulting in a possible range of 8–40.

#### 2.2.2. J-HLS-Q12

Health literacy was evaluated using the J-HLS-Q12, which showed acceptable reliability and validity in Japanese community-dwelling older adults (Kodama et al., 2023). The HLS-Q12 (Duong et al., 2017) is a shortened version of the 47-item European Health Literacy Survey Questionnaire (Sorensen et al., 2013). This questionnaire assesses health literacy through 12 items, each of which is rated on a 4-point Likert scale ranging from 1 (very easy) to 4 (very difficult). The scoring procedure involves reversing the ordinal scale values, calculating the mean of all items, and applying the following formula: score = (mean − 1) × (50/3).

### 2.3. Statistical Analysis

Descriptive statistics for the participants’ characteristics and the J-eHEALS and J-HLS-Q12 scores were reported as means and SDs for continuous variables and as frequencies and percentages for categorical variables.

Internal consistency of the J-eHEALS was assessed using Cronbach’s α (0–1), with higher values indicating greater internal consistency among items measuring the same construct. Item analysis was conducted to evaluate the contribution of each item to the overall scale, including corrected item–total correlations.

Exploratory factor analysis (EFA) was performed using maximum likelihood estimation with Promax rotation to assess the factor structure of the J-eHEALS. Sampling adequacy was examined using the Kaiser–Meyer–Olkin statistic. Factors with eigenvalues > 1.0 were considered for retention, with the parallel analysis and minimum average partial test being employed as supplementary decision criteria (Horn, 1965; Velicer, 1976). In addition to factor loadings, item communalities were computed and reported to quantify the proportion of each item’s variance accounted for by the common factor(s); communality ≥ 0.30 indicated adequate shared variance with the latent construct.

Confirmatory factor analysis (CFA) was conducted to further examine construct validity. A single-factor model with eight observed variables was specified. As the data did not meet the assumption of multivariate normality, CFA was performed using the robust maximum likelihood estimation method. The comparative fit index (CFI), goodness-of-fit index (GFI), and root mean square error of approximation (RMSEA) were calculated. CFI and GFI values > 0.90 indicated good model fit. RMSEA values < 0.08 were considered acceptable, whereas values < 0.05 denoted a close fit (Hu & Bentler, 1999). Multivariate skewness and kurtosis (Mardia’s coefficients) were calculated to assess deviation from multivariate normality and to support the use of a robust estimator. We first evaluated a baseline one-factor model without correlated error terms. Guided by the Japanese validation study by Mitsutake et al. (2011) and the semantic similarity of item content, we then specified error covariances between selected item pairs within the single factor. Only error covariances that could be theoretically justified based on overlapping wording or closely related content were retained in the final model. For the final one-factor model, we also calculated composite reliability (CR) and average variance extracted (AVE) to further evaluate reliability and convergent validity. Because the J-eHEALS was specified as a unidimensional scale, the heterotrait–monotrait ratio was not considered informative and was therefore not computed. In addition, we conducted multigroup CFA to examine measurement invariance of the single-factor model across men and women at the configural, metric, and scalar levels, using changes in the CFI and RMSEA between nested models as the decision criteria.

Criterion-related validity was evaluated by calculating Pearson’s correlation coefficient between the J-eHEALS and the J-HLS-Q12.

All analyses were performed using R software version 4.5.0 (R Foundation for Statistical Computing, Vienna, Austria), with a significance level of 5% being applied to all statistical tests. The available sample size (n = 553) exceeded the commonly recommended minimums for factor analysis and CFA, including an item-to-participant ratio > 10:1. Subgroup analyses by sex and age were regarded as exploratory and were interpreted with caution because of the smaller sample sizes in some strata.

## 3. Results

### 3.1. Participant Characteristics

A total of 553 community-dwelling older adults were included in this study, with a nearly equal distribution of men (51%, n = 282) and women (49%, n = 271). The mean age of participants was 73.5 ± 4.8 years (range, 65–90 years). Based on 5-year age intervals, 21.5% (n = 119) of the participants were aged 65–69 years, 36.9% (n = 204) were aged 70–74 years, 30.2% (n = 167) were aged 75–79 years, 9.8% (n = 54) were aged 80–84 years, and 1.6% (n = 9) were aged ≥85 years. Notably, approximately 70% of the participants were in their 70s.

### 3.2. Reliability

The internal consistency of the J-eHEALS was evaluated using Cronbach’s α. Overall, this scale showed excellent reliability (α = 0.94). The exclusion of any one of the eight items did not substantially affect overall reliability (Cronbach’s α = 0.950–0.956). The reliability remained high across participant subgroups defined by sex and age group (Cronbach’s α = 0.932–0.971; Table 1). The mean total J-eHEALS score was 20.45 ± 8.07, and the average score for each item was <3.0. Coefficients of correlation between the total J-eHEALS score and individual items ranged from 0.799 to 0.891 (Table 2).

### 3.3. Construct Validity

The adequacy of the sample for factor analysis was confirmed using the Kaiser–Meyer–Olkin measure of sampling adequacy, which yielded a value of 0.92, indicating excellent suitability. EFA of the eight J-eHEALS items using maximum likelihood estimation with Promax rotation extracted a single factor with an eigenvalue of 6.22, exceeding the threshold of 1.0 and accounting for 74.55% of cumulative variance (Table 2). The second factor had an eigenvalue of 0.63, supporting the appropriateness of a unidimensional factor structure for the J-eHEALS. All items loaded highly on this factor (range, 0.814–0.918; Table 2). Consistent with these loadings, item communalities ranged from 0.663 to 0.843, indicating adequate shared variance across items (Table 2).

**Figure 1:**
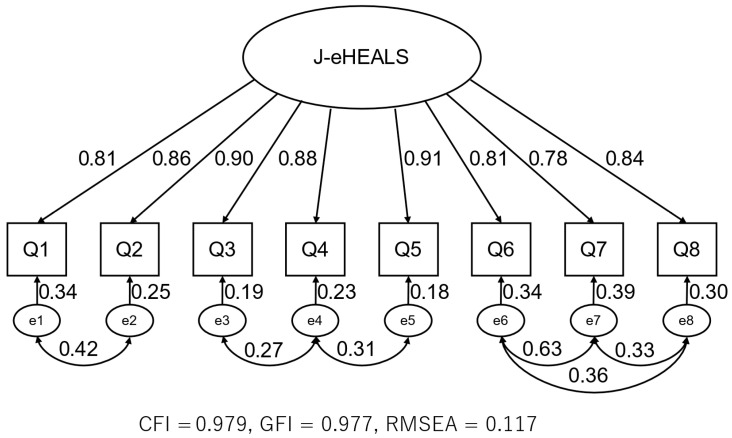
Modified model of the CFA for the J-eHEALS. Abbreviations: CFA, confirmatory factor analysis; J-eHEALS, Japanese version of the eHealth Literacy Scale; CFI, comparative fit index; GFI, goodness-of-fit index; RMSEA, root mean square error of approximation.

CFA was conducted based on a single-factor model with eight observed variables to further examine the factor structure of the J-eHEALS. Owing to the violation of normality assumptions, robust maximum likelihood estimation was performed. Mardia’s multivariate skewness and kurtosis indicated significant deviation from multivariate normality (skewness = 1009.3, *p* < 0.001; kurtosis = 70.9, *p* < 0.001), which supports the use of a robust estimator. We first tested a baseline one-factor model without correlated error terms, which yielded fit indices of GFI = 0.765, CFI = 0.883, and RMSEA = 0.228. Following the model specification reported in the Japanese validation study by Mitsutake et al. (2011) and inspection of item content, we subsequently added error covariances between the following item pairs within the single factor: Q1–Q2, Q3–Q4, Q4–Q5, Q6–Q7, Q6–Q8, and Q7–Q8. Items Q1 and Q2 both assess the awareness of available online health information resources, items Q3 and Q4 focus on searching for and using the Internet to answer health questions, and items Q4 and Q5 address the practical use of online information. Items Q6, Q7, and Q8 pertain to evaluating the quality of online health information and confidence in using it. The final model with six error covariances showed the following fit indices: GFI = 0.942, CFI = 0.977, and RMSEA = 0.117 (Figure 1).

For the final one-factor model, the CR was 0.954, and the AVE was 0.722, providing additional evidence for internal consistency and convergent validity. In the multigroup CFA stratified by sex, the configural, metric, and scalar invariance models all showed acceptable fit, and changes in the CFI between nested models were within commonly used thresholds (ΔCFI ≤ 0.01), suggesting that the factor structure and item functioning of the J-eHEALS were comparable between the sexes (Table 3). These results indicate that the correlated error terms were restricted to item pairs with closely related wording or content and were not introduced solely on the basis of modification indices.

### 3.4. Criterion-Related Validity

The J-eHEALS and J-HLS-Q12 scores exhibited a moderate positive correlation (Pearson’s r = 0.376, Spearman’s ρ = 0.396), supporting criterion-related validity. Subgroup analyses by sex and 5-year age intervals (65–69, 70–74, 75–79, and 80–84 years) yielded consistent correlation patterns (Pearson’s r = 0.324–0.453, Spearman’s ρ = 0.353–0.452; Table 4). Participants aged ≥85 years (n = 9) were excluded from the age-stratified analyses because of insufficient sample size. Subgroup analyses by sex and age were conducted as exploratory analyses.

## 4. Discussion

In the present study, we evaluated the reliability and validity of the J-eHEALS in community-dwelling older adults. Overall, the findings indicated that the J-eHEALS demonstrated excellent internal consistency and acceptable levels of factorial and criterion-related validity in this population, supporting its use as a psychometrically sound measure of perceived eHL in older Japanese adults.

The internal consistency of the J-eHEALS in the present study was excellent, with a Cronbach’s α of 0.94, which exceeded the conventional threshold of 0.70. Comparable results have been reported in older adult populations across several countries, including the United States (0.92–0.94) (Chung & Nahm, 2015; Stellefson et al., 2017), Israel (0.93) (Green, 2025), China (0.94) (Xie & Mo, 2023), Brazil (0.98) (Oliveira et al., 2022), Korea (0.90) (H. Kim et al., 2021), and Poland (0.88–0.90) (Duplaga et al., 2019). Subgroup α values by sex and age (0.932–0.971) further support the robustness of the scale. Although extremely high α values can sometimes suggest item redundancy, the corrected item–total correlations (0.799–0.891) fell within acceptable ranges, indicating sufficient distinctiveness among items. Future research should investigate whether a shortened version preserves validity and sensitivity without compromising measurement precision.

EFA supported a unidimensional factor structure, with a single factor accounting for 74.55% of the total variance (eigenvalue = 6.22). All items showed high factor loadings (0.814–0.918), consistent with the results of studies with older adult samples using other language versions of the eHEALS (Chung & Nahm, 2015; Green, 2025; H. Kim et al., 2021; Oliveira et al., 2022). CFA indicated good fit for most indices (CFI = 0.977, GFI = 0.942). The RMSEA value of 0.117 exceeded the threshold generally considered acceptable (0.08); however, this may be partly explained by the limited number of items and potential sampling effects. The RMSEA is sensitive to model misspecification and sample size, particularly in short scales such as the eHEALS (Kenny et al., 2015; Shi & Maydeu-Olivares, 2020). Taken together with the strong CFI and GFI and the unidimensional structure observed in EFA, these findings support overall factorial validity in this population. Future studies should examine whether alternative model specifications or larger samples yield a better-fitting model, thereby refining the understanding of the underlying construct of eHL among older adults.

The J-eHEALS and J-HLS-Q12 scores exhibited a moderate positive correlation (Pearson’s *r* = 0.376, Spearman’s ρ = 0.396), which aligns with the results of a previous study on older adults (*r* = 0.35) (Oliveira et al., 2022). This pattern suggests that, although related, eHL and comprehensive health literacy represent distinct constructs. Subgroup analyses by sex and age revealed consistent associations, further indicating stable performance across demographic strata.

The mean J-eHEALS score was 20.45 (SD = 8.07), indicating that the overall perceived level of eHL was moderate to slightly low. Item-level means <3.0 suggest that several participants lacked confidence or proficiency in tasks such as searching for and utilizing online health information sources. This finding aligns with reports that older adults tend to have lower eHL than that of middle-aged populations (Cherid et al., 2020; Paige et al., 2018). It also appears lower than the eHL levels reported in previous Japanese studies among younger and middle-aged adults (Mitsutake et al., 2012; Mitsutake et al., 2016; Takeda et al., 2025), which is consistent with the notion that older adults experience greater difficulties in using digital health information resources. Additionally, the high internal consistency implies that participants with relatively low confidence still interpreted and responded to the items coherently. The combination of low mean scores and strong psychometric consistency highlights both the vulnerability and measurability of eHL in this population.

Given that approximately 60% of older adults in Japan access online health information, and Internet use in this population continues to rise, the ability to accurately assess eHL has tangible relevance for public health practice. The strong reliability and validity indices observed here suggest that the J-eHEALS is a useful tool for identifying individuals or subgroups who may benefit from digital health education and support. Municipal health services, community-based programs, and primary care providers could incorporate the J-eHEALS into routine assessments and interventions aimed at reducing the digital health divide. Furthermore, policymakers may draw on these insights to inform the development of age-friendly digital platforms and educational strategies. By quantifying eHL levels, stakeholders can more effectively design interventions that align with the abilities and needs of older adults, thereby promoting equity in access to health information.

This study has some limitations. First, despite the adequate sample size (n = 553), the recruitment of participants from a single municipality may limit the generalizability of the findings to older adults in other regions or across different socioeconomic strata in Japan. Second, all measures relied on self-reports, which may be subject to recall bias or social desirability effects, potentially leading to an over- or underestimation of the participants’ actual abilities. Third, the cross-sectional design precludes assessment of responsiveness or predictive validity. Longitudinal studies should be conducted to clarify whether eHL scores change over time and whether initial scores prospectively predict digital health behaviors or clinical outcomes. Fourth, detailed indicators of health status, including medication use and specific chronic conditions, were not available in a homogenous format across all age groups and were therefore not reported. As a result, the generalizability of our findings to particular clinical subgroups (e.g., older adults with specific chronic diseases) remains uncertain. Finally, although the overall sample size was adequate for factor analysis and CFA, some age subgroups were relatively small; therefore, age-stratified analyses should be considered exploratory and interpreted with caution. In addition, the CFA model required several correlated error terms between items with similar content, which may reflect shared wording effects; future research should examine whether alternative model specifications or shortened versions of the scale can achieve adequate fit with fewer correlated errors.

Future studies should investigate whether the J-eHEALS performs consistently across different age groups (Putnick & Bornstein, 2016), digital experience levels, and health conditions and should explore changes in eHL over time or following interventions. Such studies will further clarify the utility of this scale and inform strategies to enhance digital health literacy in older populations.

## 5. Conclusions

The present study provides robust evidence supporting the reliability and factorial validity of the J-eHEALS in community-dwelling older adults in Japan. This scale showed excellent internal consistency, strong unidimensional structure, and moderate correlations with general health literacy, supporting its use as a practical and psychometrically sound tool for eHL assessment in aging populations. As digital health technologies continue to expand, measuring and addressing eHL in older adults has become crucial for promoting equitable access to online health information and supporting informed decision-making.

## Figures and Tables

**Table 1:** Cronbach’s α coefficients by sex and age group.

Variable	α
Sex	
Female	0.954
Male	0.962
Age (years)	
65–69	0.932
70–74	0.955
75–79	0.971
80–84	0.965
>85	0.960

**Table 2:** Reliability and factorial analysis of the J-eHEALS.

		Mean (SD)	Alpha If Item Deleted	Mean Item-Total Correlation	Factor Loading	Communality
Q1:	I know how to find helpful health resources on the Internet	2.86 (1.19)	0.956	0.799	0.814	0.663
Q2:	I know how to use the Internet to answer my health questions	2.70 (1.18)	0.953	0.841	0.854	0.729
Q3:	I know what health resources are available on the Internet	2.68 (1.20)	0.952	0.869	0.891	0.793
Q4:	I know where to find helpful health resources on the Internet	2.66 (1.19)	0.951	0.870	0.901	0.812
Q5:	I know how to use the health information I find on the Internet to help me	2.61 (1.16)	0.950	0.891	0.918	0.843
Q6:	I have the skills I need to evaluate the health resources I find on the Internet	2.32 (1.08)	0.954	0.834	0.848	0.719
Q7:	I can tell high-quality from low-quality health resources on the Internet	2.27 (1.06)	0.956	0.801	0.818	0.669
Q8:	I feel confident in using information from the Internet to make health decisions	2.33 (1.10)	0.953	0.838	0.858	0.736
	Total	20.45 (8.07)				
	eigenvalues = 6.220					
	% of variance explained = 74.55					

Abbreviations: SD, standard deviation; Alpha, Cronbach’s alpha; J-eHEALS, Japanese version of the eHealth Literacy Scale.

**Table 3:** Fit indices for the multigroup CFA model testing measurement invariance of the J-eHEALS across sexes.

Model	χ^2^	df	GFI	CFI	RMSEA	ΔCFI
Configural	148.04	28	0.975	0.976	0.125	—
Metric	159.52	35	0.972	0.975	0.113	−0.001
Scalar	164.35	42	0.972	0.975	0.103	<0.001

Abbreviations: CFA, confirmatory factor analysis; J-eHEALS, Japanese version of the eHealth Literacy Scale; χ^2^, chi-square; df, degrees of freedom; GFI, goodness-of-fit index; CFI, comparative fit index; RMSEA, root mean square error of approximation; ΔCFI, change in comparative fit index.

**Table 4:** Correlation coefficients between the J-eHEALS and J-HLS-Q12 by sex and age group.

Variable	r	ρ
Sex		
Female	0.375	0.395
Male	0.403	0.415
Age (years)		
65–69	0.453	0.400
70–74	0.324	0.353
75–79	0.394	0.416
80–84	0.400	0.452

Participants aged ≥85 years (n = 9) were excluded from age-stratified analyses because of insufficient sample size. Abbreviations: J-eHEALS, Japanese version of the eHealth Literacy Scale; J-HLS-Q12, Japanese version of the 12-item Health Literacy Scale; r, Pearson correlation coefficient; ρ, Spearman’s rank correlation coefficient.

## Data Availability

The data presented in this study are available on request from the corresponding author.

## Abbreviations

The following abbreviations are used in this manuscript:

AVE	Average variance extracted
CFA	Confirmatory factor analysis
CFI	Comparative fit index
CR	Composite reliability
EFA	Exploratory factor analysis
eHL	eHealth literacy
GFI	Goodness-of-fit index
J-eHEALS	Japanese version of the eHealth Literacy Scale
J-HLS-Q12	Japanese version of the 12-item Health Literacy Scale
RMSEA	Root mean square error of approximation
SD	Standard deviation
